# Fast 3D gravity and magnetic modelling using midpoint quadrature and 2D FFT

**DOI:** 10.1038/s41598-023-36525-2

**Published:** 2023-06-08

**Authors:** Xulong Wang, Jianxin Liu, Jian Li, Hang Chen

**Affiliations:** 1 School of Geosciences and Info-Physics, Central South University, Changsha, 410083 China; 2 Hunan Key Laboratory of Nonferrous Resources and Geological Hazards Exploration, Central South University, Changsha, 410083 China; 3 Key Laboratory of Metallogenic Prediction of Nonferrous Metals and Geological Environment Monitoring, Ministry of Education, Central South University, Changsha, 410083 China; 4 School of Geosciences, Boise State University, Boise, ID 83725 USA

**Keywords:** Geophysics, Mineralogy

## Abstract

To avoid the problem of the traditional methods consuming large computational resources to calculate the kernel matrix and 2D discrete convolution, we present a novel approach for 3D gravity and magnetic modelling. This method combines the midpoint quadrature method with a 2D fast Fourier transform (FFT) to calculate the gravity and magnetic anomalies with arbitrary density or magnetic susceptibility distribution. In this scheme, we apply the midpoint quadrature method to calculate the volume element of the integral. Then, the convolution of the weight coefficient matrix with density or magnetization is efficiently computed via the 2D FFT. Finally, the accuracy and efficiency of the proposed algorithm are validated by using an artificial model and a real topography model. The numerical results demonstrate that the proposed algorithm’s computation time and the memory requirement are decreased by approximately two orders of magnitude compared with the space-wavenumber domain method.

## Introduction

The efficient and accurate forward modelling of the gravity and magnetic potential fields, generated by arbitrary mass density or magnetization distribution of the 3D geological body is the foundation of the geophysical applications^1,2^. Gravity and magnetic forward modelling, which can be solved in the space or Fourier domains, is part of the potential field data processing.

The earliest numerical modelling of the potential field is mainly studied in the space domain. For the space domain forward techniques, convolution integrals satisfied by the potential fields can be directly calculated by analytical solution^3–8^, Gaussian quadrature method^9–11^, Cauchy-type integral method^12–14^ or multilevel fast multipole method^1,15^. However, whether performed by analytical formulea or numerical integration, several calculations are required for every subdivided volume cell^16^. Moreover, the discrete convolution of the weight coefficients with density or magnetization takes much time. Therefore, 3D gravity and magnetic numerical modelling suffer from a prohibitive computational cost, especially when the number of observation sites and source elements are huge^14,15^.

Another method to evaluate the convolution integrals is transformed into the Fourier domain. Many researchers have considered fast computation of integral kernel, including the calculation of the integral in the Fourier domain^17–20^ and by discretization of the operator and calculation in the space domain^21–25^. Then, Li et al.^17^ and Dai et al.^18^ developed the space-wavenumber domain method based on 2D Gauss-FFT^26^ to calculate gravity and magnetic anomalies. Their research mainly focus on reducing the boundary effect of the standard FFT in the Fourier domain, which significantly improving the accuracy.

Forward modelling for geophysics kernels has utilized the FFT in a variety of situations. Vogel^27^ first applied the FFT algorithm to the integral kernel where observations and dipoles are aligned on a regularly-spaced grid. For a multilayer model, Zhang and Wong^21^ introduced the use of the Block-Toeplitz-Toeplitz-Block (BTTB) structure for fast 3D gravity forward modelling and inversion. Wu^22^ combined the Gaussian quadrature and FFT algorithm to develop a fast method for complex 3D gravity forward modelling. Subsequently, Chen and Liu^23^ used an analytical expression to calculate the weight coefficients, but only for gravity case. Yuan et al.^25^ achieved the magnetic forward modelling based BTTB matrix. Hogue et al.^24^ discussed the properties of the coefficient matrix of the gravity and magnetic potential field. However, the majority of previous work apply the analytical soulution or Gaussian quadrature method to caluculate the kernel martrix which hit a computational cost and memory requirement constraint, rendering them prohibitive in scenarios with a large number of mesh elements.

In this study, we propose a novel method to solve large-scale gravity and magnetic potential field forward problems swiftly. In this scheme, we introduce an approach to explore the symmetric structures of the coefficient matrix for the gravity and magnetic kernel. To reduce the computation time, the midpoint quadrature^28,29^ is used to calculate the coefficient matrix, and the 2D FFT is applied to achieve the fast discrete convolution of the coefficient matrix with the density or magnetization function. The accuracy and efficiency of our algorithm are examined using a synthetic model and a realistic topography model.

## Basic theory

### Statement of problem

For general mass density or magnetization distribution, the integral response of both gravity and magnetic signals can be expressed as the convolution of the Green’s function $G({\textbf{r}},{\textbf{r}}^{\prime })$ with the source function $f({\textbf{r}}^{\prime })$^30^

1 $$\begin{aligned} h({\textbf{r}})=c\int _{\Omega } G({\textbf{r}},{\textbf{r}}^{\prime }) f({\textbf{r}}^{\prime }) dv, \end{aligned}$$

which can be further simplified as

2 $$\begin{aligned} h({\textbf{r}})=J({\textbf{r}},{\textbf{r}}^{\prime })f({\textbf{r}}^{\prime }) dv, \end{aligned}$$

where ${\textbf{r}}$ is the field coordinates, ${\textbf{r}}^{\prime }$ is the source coordinates, $h({\textbf{r}})$ represents the response of the gravity or magnetic signals, $J({\textbf{r}},{\textbf{r}}^{\prime })=cG({\textbf{r}},{\textbf{r}}^{\prime })$ is the weight coefficients matrix. For the gravity forward problem, $c=\gamma$, $f({\textbf{r}}^{\prime })=\rho ({\textbf{r}}^{\prime })$, $\gamma$ is the gravitational constant, $\rho ({\textbf{r}}^{\prime })$ is the density distribution function. For the magnetic forward problem, $c=\mu$, $f({\textbf{r}}^{\prime })={\textbf{M}}({\textbf{r}}^{\prime })$, $\mu$ is the permeability of vacuum, ${\textbf{M}}({\textbf{r}}^{\prime })$ is the intensity of the magnetization distribution function.

### Numerical integration of 3D integral

The computational burden of evaluating element integral by an analytical solution^7,31^ or numerical integration^14,15^ is pretty large, even if the density or magnetization profile of the cell is as simple as constant. To solve it, we introduce the midpoint quadrature method^28,29^ to calculate the volume element of the integration by multiplying the value of the midpoint with the length of the integration interval. In this section, $J({\textbf{r}},{\textbf{r}}^{\prime })$ can be computed by applying the midpoint quadrature method. The model space $[x_{1},x_{L}]\times [y_{1},y_{M}]\times [z_{1},z_{N}]$ can be discretized into $L\times M\times N$ grid points. And, we assume that the mass density or magnetization of each grid point is constant. Therefore, for the fields point $(x_{l}, y_{m}, z_{0})$ on the 2D horizontal grid, the general 3D convolution problem can be written as

3 $$\begin{aligned} \begin{aligned}{}&h(x_{l}, y_{m}, z_{0})=\sum _{{i}=1}^{L} \sum _{j=1}^{M} \sum _{k=1}^{N} J(x_{l}-x_{i}^{\prime },y_{m}- y_{j}^{\prime },z_{0}- z_{k}^{\prime }) f(x_{i}^{\prime }, y_{j}^{\prime }, z_{k}^{\prime }),\\&\qquad \qquad \qquad l=1,2,\cdots ,L, \quad m=1,2,\cdots ,M, \end{aligned} \end{aligned}$$

where $(x_{i}^{\prime }, y_{j}^{\prime }, z_{k}^{\prime })$ are the coordinate of the source point (*i*, *j*, *k*), $f(x_{i}^{\prime }, y_{j}^{\prime }, z_{k}^{\prime })$ is the density or magnetization of the source point. Equation (3) can be further expressed as

4 $$\begin{aligned} \begin{aligned}{}&h(x_{l}, y_{m}, z_{0})=\sum _{k=1}^{N} h^{k} (x_{l}, y_{m}, z_{0})\\ {}&\qquad \qquad \qquad =\sum _{k=1}^{N} \left( \sum _{l=1}^{L} \sum _{j=1}^{M} J(x_{l}-x_{i}^{\prime },y_{m}- y_{j}^{\prime },z_{0}- z_{k}^{\prime }) f(x_{i}^{\prime }, y_{j}^{\prime }, z_{k}^{\prime }) \right) , \end{aligned} \end{aligned}$$

let

5 $$\begin{aligned} h(x_{l}, y_{m}, z_{0})=\sum _{k=1}^{N} h^{k} (x_{l}, y_{m}, z_{0}) =\sum _{k=1}^{N} J_{LM}^{k} f_{LM}^{k}, \end{aligned}$$

where

6 $$\begin{aligned} J_{LM}=\mu _{l} \nu _{m} \omega _{n} G(x_{l}-x_{i}^{\prime },y_{m}- y_{j}^{\prime },z_{0}- z_{k}^{\prime }), \end{aligned}$$

$(\mu _{l}, \nu _{m}, \omega _{n})$ are the quadrature weights, $\mu _{l}=\dfrac{x_{L}-x_{1}}{L}$, $\nu _{m}=\dfrac{y_{M}-y_{1}}{M}$, $\omega _{n}=\dfrac{z_{N}-z_{1}}{N}$. Taking gravity field’s Green function as an example, the weight coefficient of the formula (6) can be expressed as

7 $$\begin{aligned} \begin{aligned}{}&J_{LM}=\mu _{l} \nu _{m} \omega _{n} G(x_{l}-x_{i}^{\prime },y_{m}- y_{j}^{\prime },z_{0}- z_{k}^{\prime })\\&\qquad =\mu _{l} \nu _{m} \omega _{n} \gamma \dfrac{z_{0}- z_{k}^{\prime }}{[(x_{l}-x_{i}^{\prime })^{2} +(y_{m}- y_{j}^{\prime } )^{2}+ (z_{0}- z_{k}^{\prime })]^{3/2} }, \end{aligned} \end{aligned}$$

Similarly, we can obtain Green’s function the weight coefficients for gravity and magnetic anoamly quantities.

### Acceleration technique for 2D convolution

When the observation point coincides with the horizontal projection of the centroid of the splitting point masses, the weight coefficient matrix $J_{LM}$ is a block Toeplitz matrix^23^. Due to a large number of repeated matrix elements, the full matrix can be obtained by calculating only some elements. Thus, it can effectively reduce the calculation time of the weight coefficient matrix. Finally, the convolution problem of the weight coefficient matrix $J_{LM}$ with $f_{LM}$ for Eq. (5) can be achieved using the 2D FFT.

According to the convolution theorem:

8 $$\begin{aligned} F_{2D}(g)=F_{2D}(f \otimes \rho )=F_{2D}(f).*F_{2D}(\rho ), \end{aligned}$$

where $F_{2D}$ denotes the 2D discrete Fourier transform operators; ‘$\otimes$’ denotes the 2D multiplication operator; ‘$.*$’ is the dot multiplication operator. It can be found that the convolution of $J_{LM}$ and $f_{LM}$ is converted to the product of the Fourier domain with the help of the 2D FFT technique. Applying the convolution theorem, Eq. (5) can be computed by

9 $$\begin{aligned} {\tilde{h}}^{k}=F_{2D}({\tilde{J}}^{k}).*F_{2D}({\tilde{f}}^{k}), \end{aligned}$$

where

10 $$\begin{aligned} \begin{aligned}{}&{\tilde{J}}^{k}={\tilde{J}}_{l-i,m-j}^{k}= \left[ \begin{array}{ccccccc} J_{0,0} &{} \cdots &{} J_{0,M-1} &{} 0 &{} J_{0,1-M}&{} \cdots &{} J_{0,1}\\ \vdots &{} \ddots &{} \vdots &{} \vdots &{} \vdots &{} \ddots &{} \vdots \\ J_{L-1,0} &{} \cdots &{} J_{L-1,M-1} &{} 0 &{} J_{L-1,1-M}&{} \cdots &{} J_{L-1,1}\\ 0 &{} \cdots &{} 0 &{} 0 &{} 0 &{} \cdots &{} 0 \\ J_{1-L,0} &{} \cdots &{} J_{1-L,M-1} &{} 0 &{} J_{1-L,1-M}&{} \cdots &{} J_{1-L,1}\\ \vdots &{} \ddots &{} \vdots &{} \vdots &{} \vdots &{} \ddots &{} \vdots \\ J_{1,0} &{} \cdots &{} J_{1,M-1} &{} 0 &{} J_{1,1-M}&{} \cdots &{} J_{1,1}\\ \end{array} \right] , \end{aligned} \end{aligned}$$

and then Eq. (10) can be further denoted as

11 $$\begin{aligned} {\tilde{J}}^{k}= \left[ \begin{array}{ccccccc} {\textbf{t}}_{L \times M}^{11} &{} {\textbf{t}}_{L \times M}^{12} \\ {\textbf{t}}_{L \times M}^{21} &{} {\textbf{t}}_{L \times M}^{22} \\ \end{array} \right] , \end{aligned}$$

and

12 $$\begin{aligned} {\tilde{f}}^{k}= \left[ \begin{array}{ccccccc} {\textbf{f}}_{L \times M} &{} {\textbf{0}}_{L \times M} \\ {\textbf{0}}_{L \times M} &{} {\textbf{0}}_{L \times M} \\ \end{array} \right] . \end{aligned}$$

The final source matrix $f_{2L \times 2M}$ is obtained by filling the original source matrix $f_{L \times M}$ with zero element, which is equal to the size of kernel matrix.

Appling the 2D inverse Fourier transform on Eq. (9), the gravity anomaly in the space domain can be obtained

13 $$\begin{aligned} h=\sum _{k=1}^{N} h^{k} \lfloor F_{2D}^{-1}({\tilde{h}} ^{k}) \rfloor _{L \times M}, \end{aligned}$$

where $F_{2D}^{-1}$ denotes the 2D inverse discrete Fourier transform operators. $\lfloor \rfloor _{L \times M}$ means to take the first *L* row and *M* column elements of the matrix. Finally, the gravitational anomaly is obtained by accumulating the influence of the *N* layer source on the observation height. Repeating the above process, we can also easily get the magnetic anomaly.

## Results

In this section, we examine the proposed method by presenting two different kinds of numerical experiments. At first, we use a benchmark scenario model^32,33^ to verify the proposed algorithm and evaluate the performance of the proposed algorithm by comparing it with existing implementation algorithms. Then, we apply the proposed approach to a local real topographic model in New Zealand to demonstrate the practical application. All the numerical experiments are implemented on a laptop with Intel Core i5-10210U 1.6 GHz and 8 GB of RAM.

### Performance comparison with existing implementations

**Figure 1:**
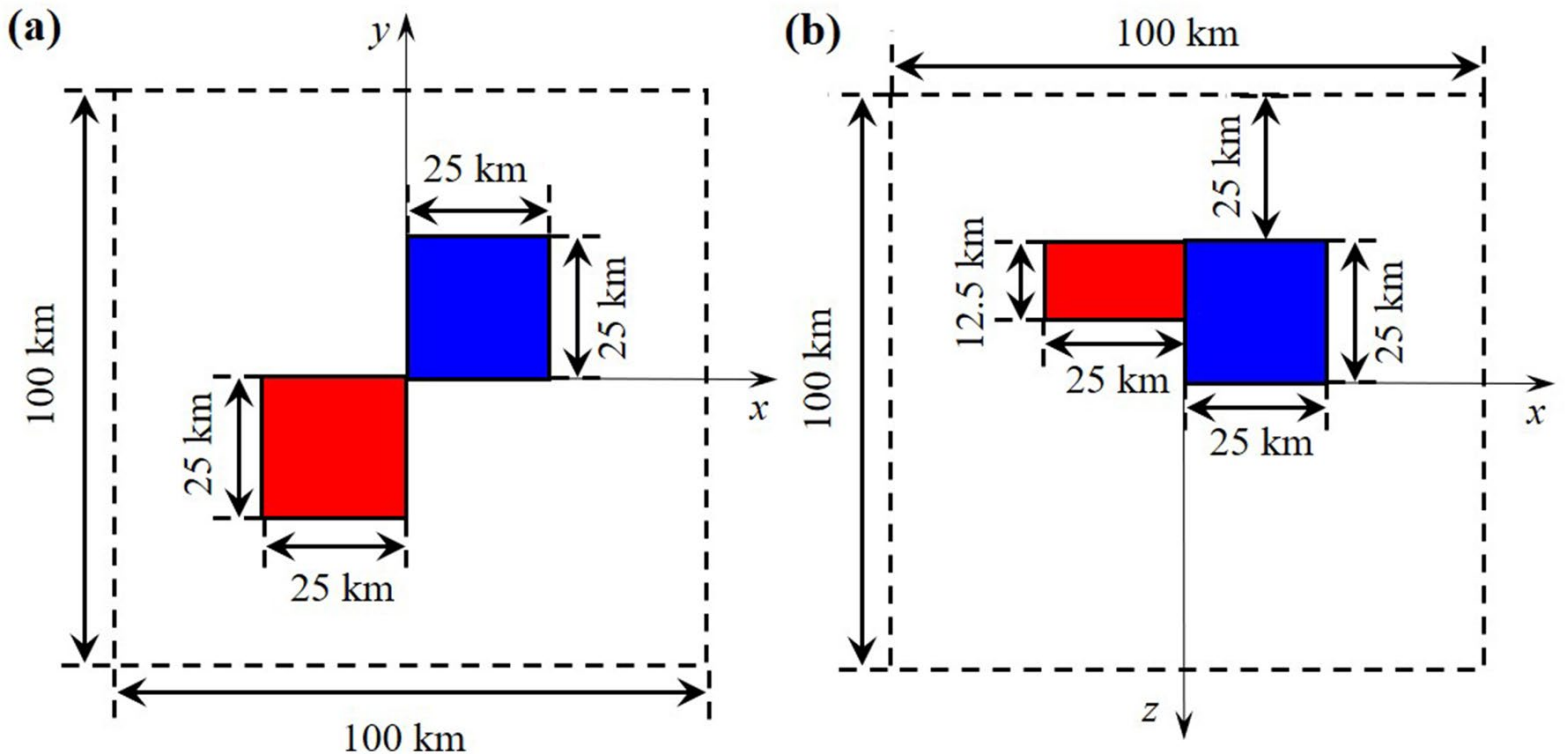
The combined prism density or magnetization model. Red: 1000 kg/m$^{3}$ or 0.03 SI, blue: − 1000 kg/m$^{3}$ or 0.01 SI^32^. (**a**) A horizontal slice of the model at z = 0 km depth and (**b**) the profile section along y = 0 km.

As shown in Fig. 1, the benchmark model is used to verify the correctness and evaluate the performance of the gravity and magnetic forward modelling code. The model setup is identical to that used in Pan et al.^32^, with a modelling domain of 100 km $\times$ 100 km $\times$ 100 km , which contains two cubic bodies. The model region is discretized into 128 $\times$ 128 $\times$ 128 cells with an equal interval of 781.25 m in three directions. The number of observation points is 128 $\times$ 128 on a horizontal observation plane with a height of 12.5 km.

**Figure 2:**
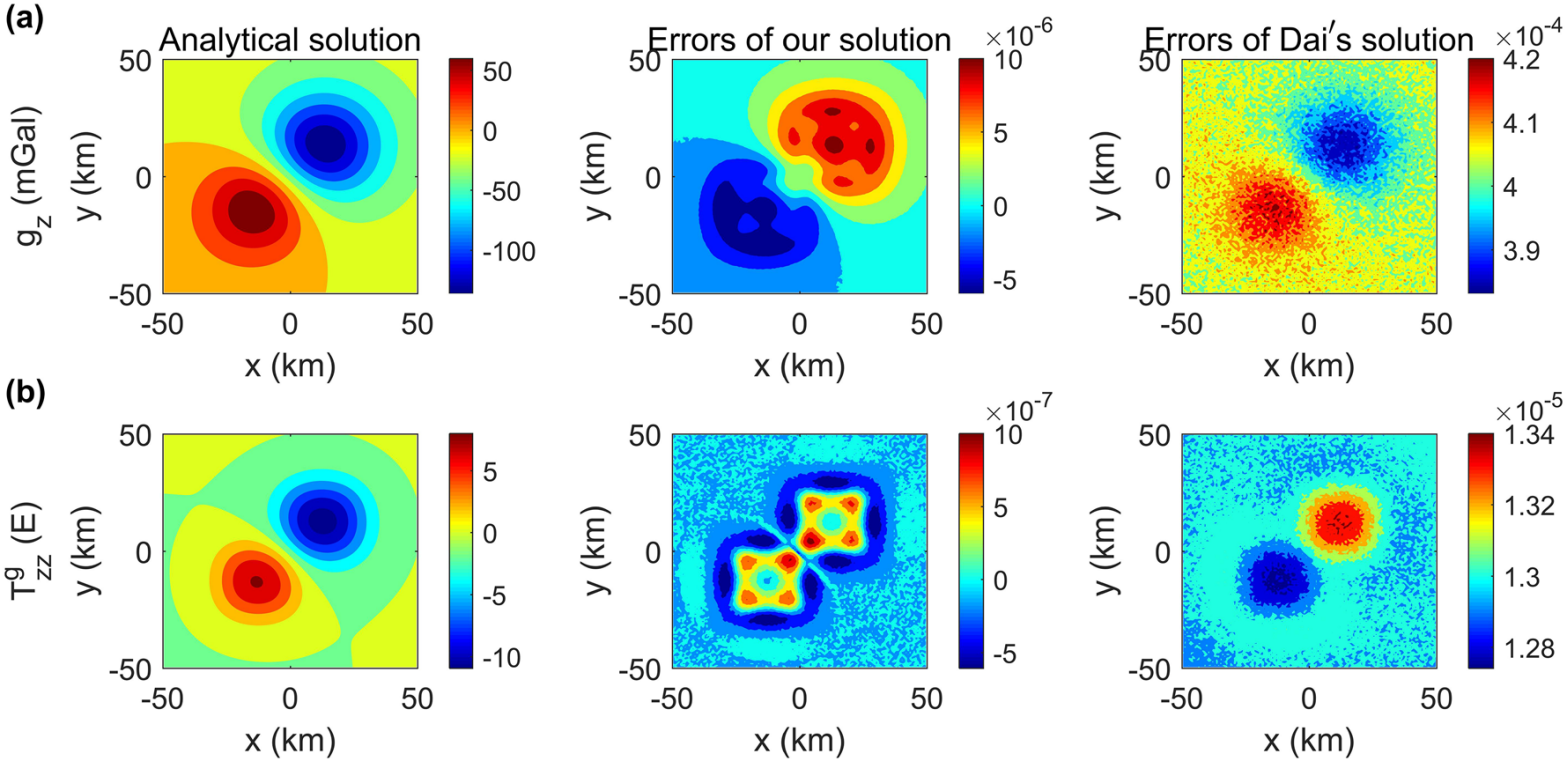
Comparison of the absolute errors between our solution and the method of Dai et al.^18^ for the gravity field $g_{z}$ and gravity gradient $T_{zz}^{g}$ caused by the synthetic model shown in Fig. 1 on the plane of z = 12.5 km.

**Figure 3:**
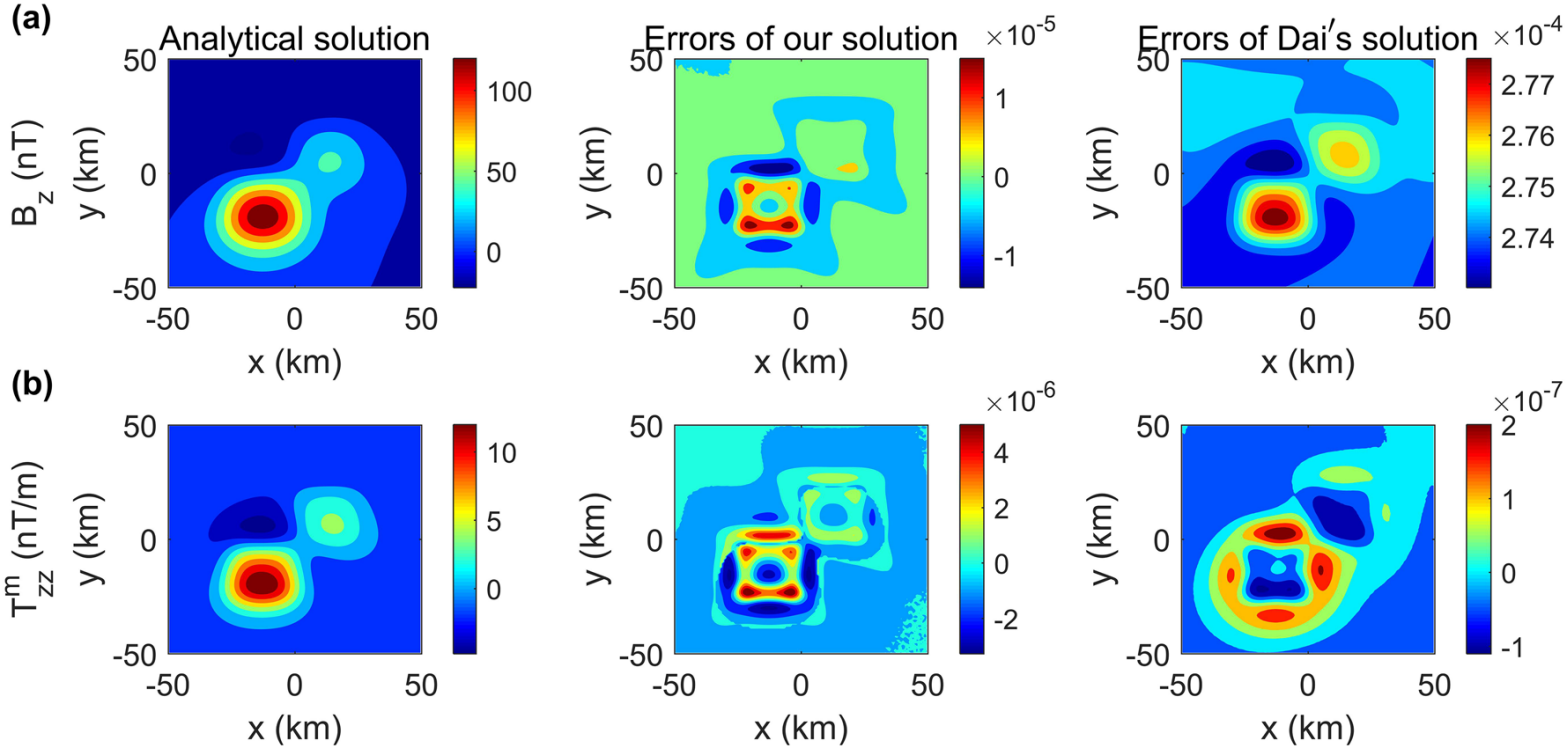
Comparison of the absolute errors between our solution and the method of Dai et al.^18^ for the magnetic field $B_{z}$ and magnetic gradient $T^{m}_{zz}$ caused by the synthetic model shown in Fig. 1 on the plane of z = 12.5 km.

The forward results in comparison with the analytical solution^30^ are shown in Figs. 2 and  3. For the gravity case, the maximum errors for the gravity field $g_{z}$ and gravity gradient $T_{zz}^{g}$ are 1.07 × 10$^{-5}$ mGal and 1.06 × 10$^{-6}$ E, respectively. For the magnetic case, the maximum errors for the magnetic field $B_{z}$ and magnetic gradient $T^{m}_{zz}$ are 1.7 × 10$^{-5}$ nT and 5.91 × 10$^{-6}$ nT/m, respectively. The trivial difference between the numerical results and analytical solution for all data types obviously verifies the accuracy of the proposed algorithm.

To evaluate the numerical performance of the proposed method, the forward results are compared with the space-wavenumber domain method^18^, which transforms the 3D integral equation methods into a 1D integral with different wavenumbers. In addition to the method mentioned^18^, we also compare our method with the compact difference scheme for solving the Posson’s equation^32^.

**Figure 4:**
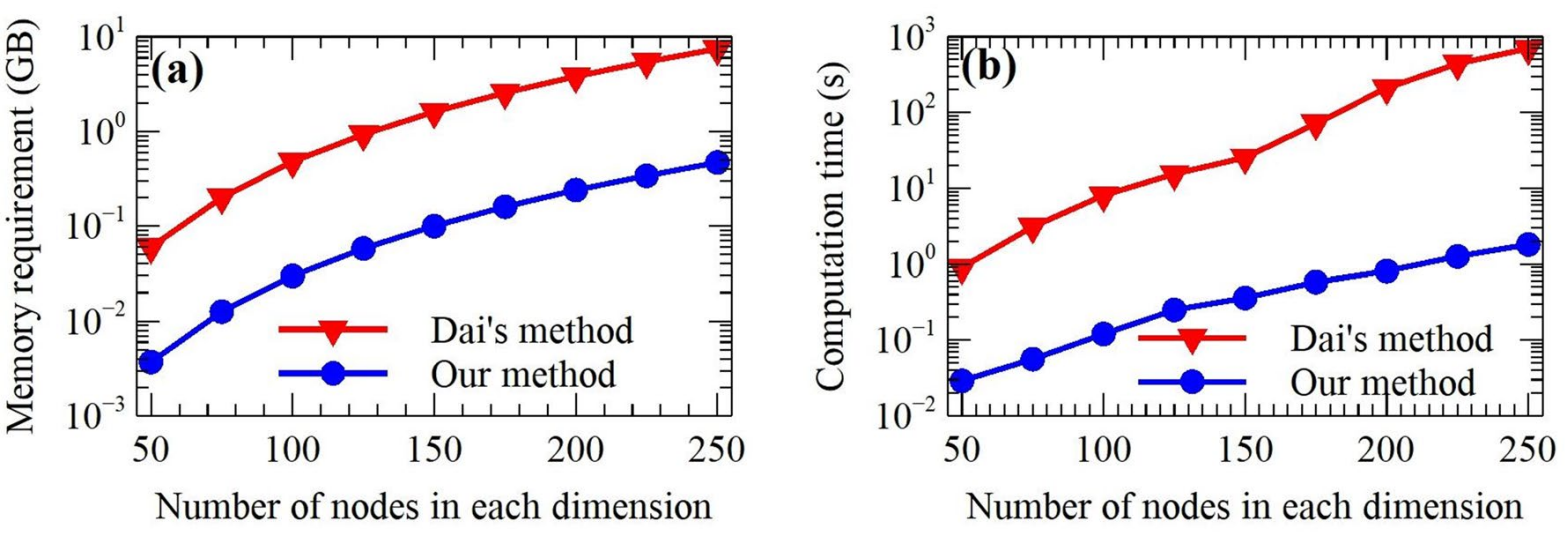
Comparison of the computation time and memory requirement of the proposed method and Dai et al.^18^.

**Table 1:** Comparison of the computational performance of different methods.

Methods	Time (s)	Maximum absolute error (E)	Rrms ($\%$)	Computing resources
Pan et al.’s method	31.0	9.25 × 10$^{-4}$	9.2 × 10$^{-3}$	2.6 GHz Intel Core i7 and 16 GB
Dai et al.’s method	19.48	1.34 × 10$^{-5}$	4.32 $\times$ 10$^{-4}$	1.6 GHz Intel Core i5 and 8 GB
Our method	0.25	1.06 $\times$ 10$^{-6}$	7.89 $\times$ 10$^{-6}$	1.6 GHz Intel Core i5 and 8 GB

The gravity field $g_{z}$ and gravity gradient $T_{zz}^{g}$ of the analytical solutions are shown in the first columns of Fig. 2. The absolute differences of our method and the space-wavenumber domain method with 8 gauss nodes^18^ are shown in the second and third columns of Fig. 2, respectively. For the gravity field $g_{z}$ and the gravity gradient $T_{zz}^{g}$, we can find that the differences of our method are two orders of magnitude lower than those of Dai et al.^18^. Taking $T_{zz}^{g}$ as an example, the execution time, rrms, maximum absolute error and computing resources for the different methods are shown in Table 1. The maximum error and the relative root mean square error (Rrms) of our method is 1.06 × 10$^{-6}$ E and 7.89 × 10$^{-6}$ $\%$ , which are much lower than Dai et al.^18^ (1.34 × 10$^{-5}$ E and 4.32 × 10$^{-4}$ $\%$) and Pan et al.^32^ (9.25 × 10$^{-4}$ E and 9.2  × 10$^{-3}$ $\%$). In addition, the calculation time of the proposed method only required 0.25 s, whereas 19.48 s and 31.0 s were required for the method of Dai et al.^18^ and Pan et al.^32^, respectively. Our method has a speedup ratio of 78 and 124 over the Dai et al.^18^ and Pan et al.^32^ methods, respectively. It can be seen that our proposed method has higher computational accuracy and efficiency.

We also study the execution time and memory requirement in cases of different model sizes. The model is discretized into different small cells, which are from 50 $\times$ 50 $\times$ 50 cells to 250 $\times$ 250 $\times$ 250 cells. As illustrated in Fig. 4b, the computation time of our method increases from 0.029 s (50 $\times$ 50 $\times$ 50) to 1.81 s (250 $\times$ 250 $\times$ 250). By contrast, the method of Dai et al.^18^ spends 0.9 s and 699.26 s for the same condition. It can be evident that the computation time of both approaches increases exponentially with the increase of the mesh as shown in Fig. 4b. However, the computation time of our method is more gently compared with Dai et al.^18^. Moreover, our method requires only 1/16 of the computation memory of Dai et al.^18^ in Fig. 4a. So, it can be further considered that our method is more favor for 3D large-scale gravity and magnetic forward problems.

### Topography effect on airborne data

**Figure 5:**
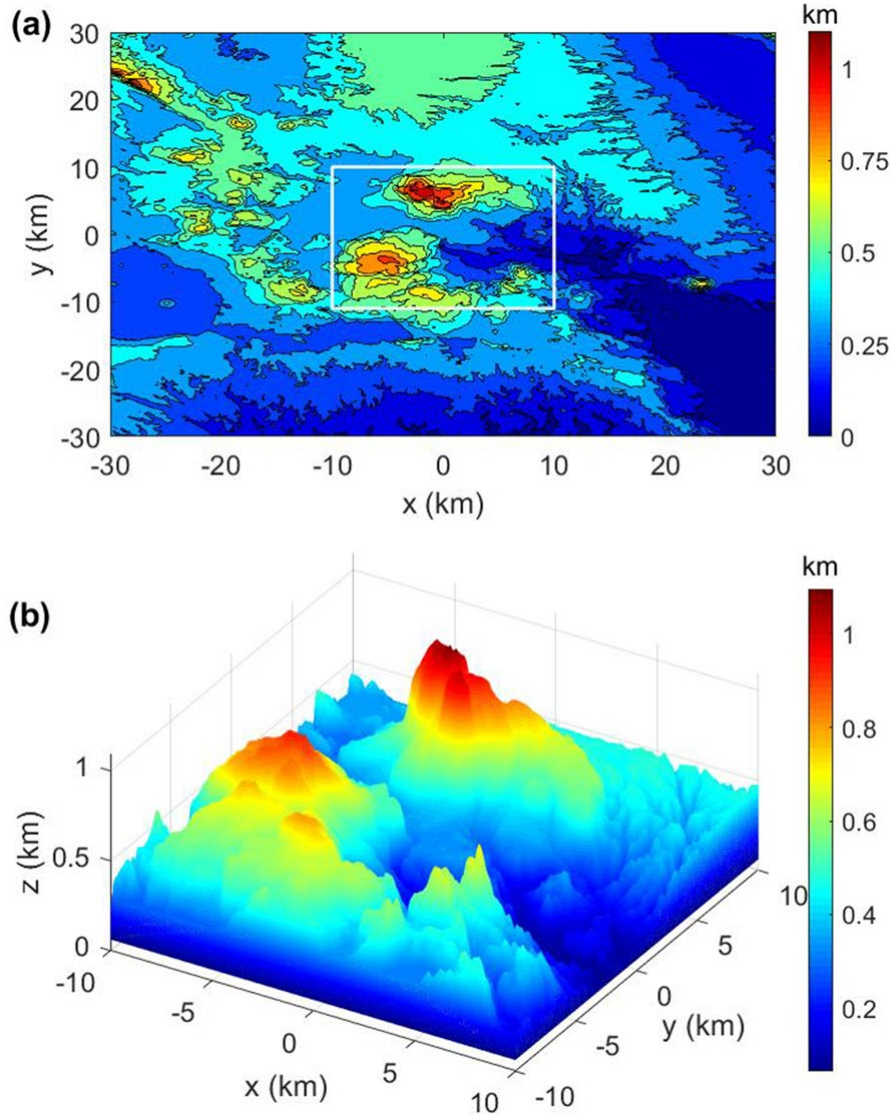
(**a**) Map of DEM digital elevation data. (**b**) The white frame marks the observation area containing the most complex areas of undulating terrain.

Figure 5 shows the final testing model, which derived from a digital elevation model (DEM) of Rotorua city, New Zealand, with significant terrain. Our motivation is to set up a rugged topography model to investigate the effect of topography on airborne gravity and magnetic data. The DEM data with a spatial resolution 3$^{\prime \prime }$ $\times$ 3$^{\prime \prime }$, can be obtained from the http://dwtkns.com/srtm/. The DEM data are resampled with a spacing of 50 m in the horizontal directions. The area’s maximum elevation of the undulating terrain is 1091 m, and the lowest is 3.2 m. Thus, the rugged topography model can be built by 400 $\times$ 400 $\times$ 100 cells. For all elements below the topographic ground surface, we assign a constant value of 2760 kg m$^{-3}$. The magnetic field strength is 53861 nT, and the magnetic declination and inclination are 20.9$^{\circ }$ and − 63.5$^{\circ }$, respectively. The local magnetic data can be obtained from the Magnetic Declination website (Magnetic declination, n.a.). The magnetic susceptibility of the region is taken as 0.02 SI. We set up a horizontal computational plane with 400 $\times$ 400 observation sites that cover the entire area at a local height of 2000 m to imitate the airborne gravity and magnetic survey.

**Figure 6:**
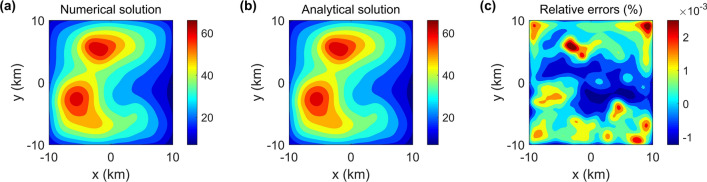
Numerical solution, analytical solution^33^ and their relative errors.

**Figure 7:**
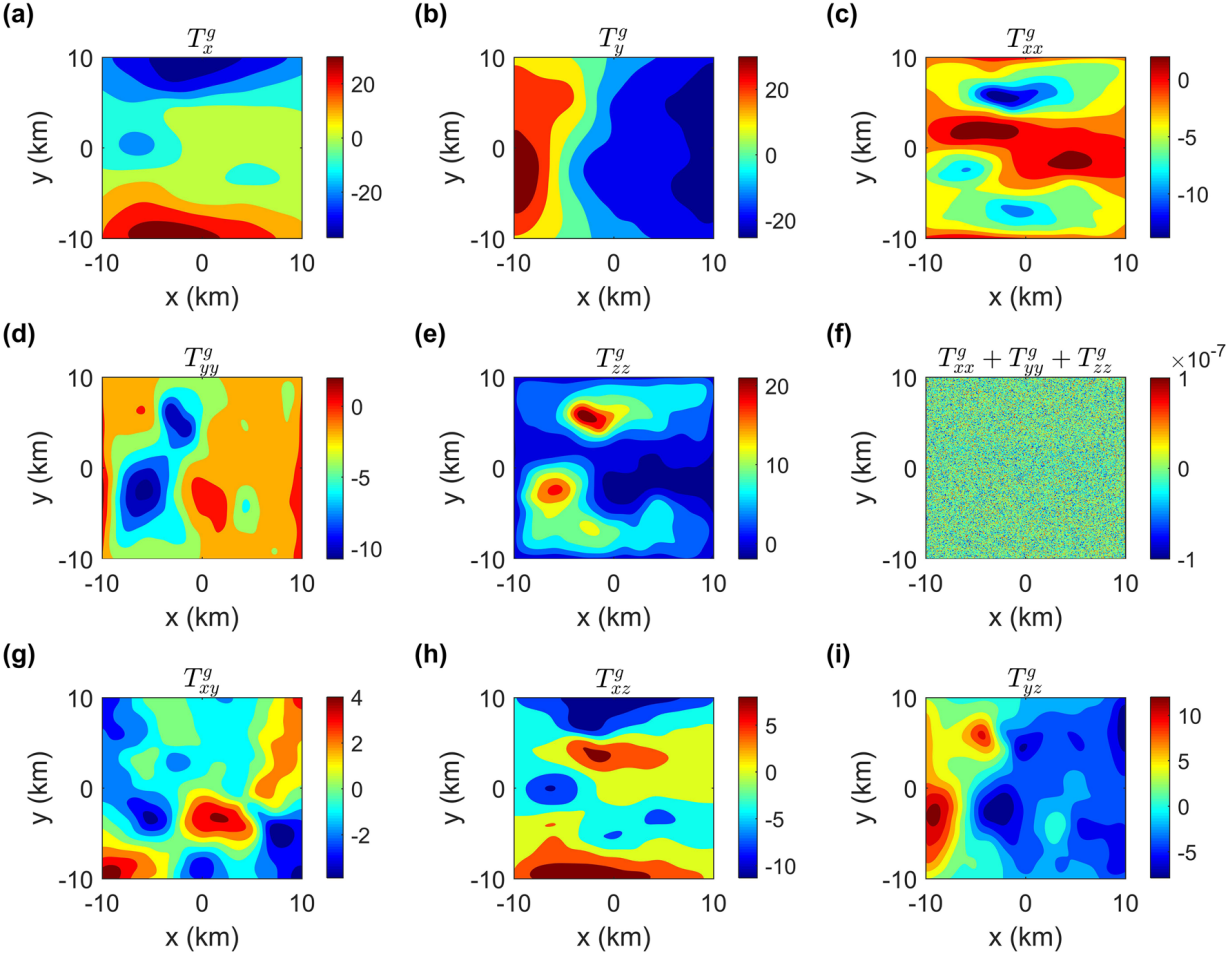
Numerical solutions of the gravity fields and gravity gradient tensors.

**Figure 8:**
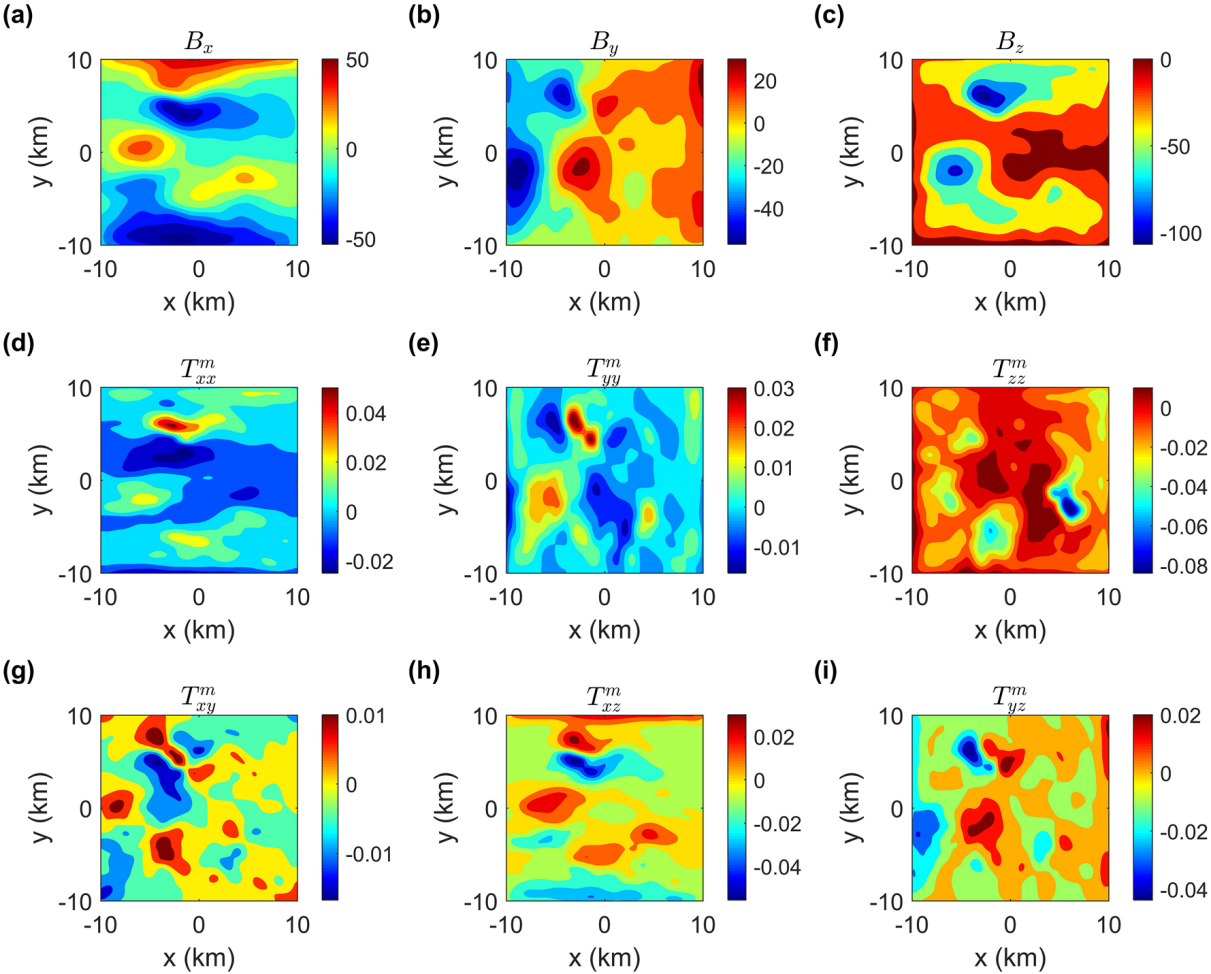
Numerical solutions of the magnetic fields and magnetic gradient tensors.

We calculate the vertical gravity to evaluate the accuracy and efficiency of the proposed method based on the terrain mode as shown in Fig. 5b. Figure 6 compares the vertical gravity computed using our method with the analytical solution based on cell merging and parallel computing^33^, and both solutions are in excellent agreement. The maximal relative error of the two methods is less than 2.2 × 10$^{-3}$ $\%$. Additionally, compared to the 46,779.56 s required by the analytical solution, our method only took 2.56 s. Our solution has a speedup ratio of 18,273 over the analytical solution based on cell merging and parallel computing. The excellent agreement of the two solutions, as well as the considerable speedup clearly demonstrate the better performance of our method for tackling complicated topography model.

In addition, we also compute the gravity fields (Fig. 7a,b), the gravity gradient tensors (Fig. 7c–i), the magnetic fields (Fig. 8a–c) and the magnetic gradient tensors (Fig. 8d–i) caused by the terrain model. From Fig. 7f, we can see that the maximum value of $T_{xx}^{g}+T_{yy}^{g}+T_{zz}^{g}$ is less than 1.0 × 10$^{-7}$ E, which is approximately equal to 0. Obviously, it again demonstrates that our method is suitable for a complex topography model.

## Discussion

In this article, we propose an efficient approach by utilizing the midpoint quadrature for computing the weight coefficient matrix and the 2D FFT method to directly calculate gravity and magnetic anomalies. We evaluate its precision and effectiveness using an artificial model and a real topography model. The results show that our method can almost ignore the effect near the boundary compared with the traditional Fourier domain method based on Gauss-FFT. At the same time, this approach not only greatly improves efficiency, but also requires much less computational memory. The results show the great potential of our method to be applicable to 3D fast inversion of gravity and magnetic data in a large-scale realistic terrain model.

## Data Availability

The datasets generated during and analysed during the current study are available from the corresponding author on reasonable request.

## References

[1] Casenave F, Metivier L, Pajot-Metivier G, Panet I (2016). Fast computation of general forward gravitation problems. J. Geodesy.

[2] Dai, S., Zhao, D., Wang, S., Xiong, B. & Chen, Q. Three-dimensional numerical modeling of gravity and magnetic anomaly in a mixed space-wavenumber domain. *Geophysics**84*, 41–54 (2019).

[3] Takin M, Talwani M (1966). Rapid computation of the gravitation attraction of topography on a spherical earth. Geophys. Prospect..

[4] Nagy D (1966). The gravitational attraction of a right rectangular prism. Geophysics.

[5] Okabe M (1979). Analytical expressions for gravity anomalies due to homogeneous polyhedral bodies and translations into magnetic anomalies. Geophysics.

[6] Li X, Chouteau M (1998). Three-dimensional gravity modeling in all space. Surv. Geophys..

[7] Nagy D, Papp G, Benedek J (2000). The gravitational potential and its derivatives for the prism. J. Geodesy.

[8] Ren Z, Zhong Y, Chen C, Tang J, Pan K (2018). Gravity anomalies of arbitrary 3d polyhedral bodies with horizontal and vertical mass contrasts up to cubic ordergravity for 3d polyhedral mass body. Geophysics.

[9] Ku CC (1977). A direct computation of gravity and magnetic anomalies caused by 2-and 3-dimensional bodies of arbitrary shape and arbitrary magnetic polarization by equivalent-point method and a simplified cubic spline. Geophysics.

[10] Asgharzadeh M, Von Frese R, Kim H, Leftwich T, Kim J (2007). Spherical prism gravity effects by Gauss-Legendre quadrature integration. Geophys. J. Int..

[11] Zhong Y (2019). A new method for gravity modeling using tesseroids and 2d Gauss-Legendre quadrature rule. J. Appl. Geophys..

[12] Cai H, Zhdanov MS (2014). Modeling and inversion of magnetic anomalies caused by sediment-basement interface using three-dimensional cauchy-type integrals. IEEE Geosci. Remote Sens. Lett..

[13] Cai, H. & Zhdanov, M. S. Joint inversion of gravity and magnetotelluric data for the depth-to-basement estimation. *IEEE Geosci. Remote Sens. Lett.* **PP**, 1–5 (2017).

[14] Mohammadi N, Motavalli-Anbaran S-H, Ebrahimzadeh Ardestani V (2021). Improved 3D Cauchy-type integral for faster and more accurate forward modeling of gravity data caused by basement relief. Pure Appl. Geophys..

[15] Ren Z, Tang J, Kalscheuer T, Maurer H (2017). Fast 3-D large-scale gravity and magnetic modeling using unstructured grids and an adaptive multilevel fast multipole method. J. Geophys. Res. Solid Earth.

[16] Zhdanov MS, Liu X (2013). 3-D Cauchy-type integrals for terrain correction of gravity and gravity gradiometry data. Geophys. J. Int..

[17] Li K (2018). Fast 3D forward modeling of the magnetic field and gradient tensor on an undulated surface. Appl. Geophys..

[18] Dai S, Chen Q, Li K, Ling J (2022). The forward modeling of 3d gravity and magnetic potential fields in space-wavenumber domains based on an integral method. Geophysics.

[19] Wang X (2021). Fast numerical simulation of 2d gravity anomaly based on nonuniform fast Fourier transform in mixed space-wavenumber domain. J. Appl. Geophys..

[20] Wang X, Zhao D, Liu J, Zhang Q (2022). Efficient 2D modeling of magnetic anomalies using NUFFT in the Fourier domain. Pure Appl. Geophys..

[21] Zhang Y, Wong Y (2015). BTTB-based numerical schemes for three-dimensional gravity field inversion. Geophys. J. Int..

[22] Wu L (2018). Efficient modeling of gravity fields caused by sources with arbitrary geometry and arbitrary density distribution. Surv. Geophys..

[23] Chen L, Liu L (2019). Fast and accurate forward modelling of gravity field using prismatic grids. Geophys. J. Int..

[24] Hogue JD, Renaut RA, Vatankhah S (2020). A tutorial and open source software for the efficient evaluation of gravity and magnetic kernels. Comput. Geosci..

[25] Yuan Y (2022). Fast and high accuracy 3D magnetic anomaly forward modeling based on BTTB matrix. Chin. J. Geophys..

[26] Wu L, Tian G (2014). High-precision Fourier forward modeling of potential fields. Geophysics.

[27] Vogel CR (2002). Computational Methods for Inverse Problems.

[28] Dragomir SS (2000). On the midpoint quadrature formula for mappings with bounded variation and applications. Kragujevac J. Math..

[29] Jeyakarthikeyan PV, Subramanian G, Yogeshwaran R (2017). An alternate stable midpoint quadrature to improve the element stiffness matrix of quadrilaterals for application of functionally graded materials (FGM). Comput. Struct..

[30] Blakely RJ (1996). Potential Theory in Gravity and Magnetic Applications.

[31] Fukushima T (2020). Speed and accuracy improvements in standard algorithm for prismatic gravitational field. Geophys. J. Int..

[32] Pan K (2021). Three-dimensional forward modelling of gravity field vector and its gradient tensor using the compact difference schemes. Geophys. J. Int..

[33] Chen T, Zhang G (2018). Forward modeling of gravity anomalies based on cell mergence and parallel computing. Comput. Geosci..

